# Dataset of Indonesian women’s reproductive, high-fat diet and body mass index risk factors for breast cancer

**DOI:** 10.1016/j.dib.2021.107107

**Published:** 2021-04-29

**Authors:** Ricvan Dana Nindrea, Elly Usman, Yusticia Katar, Ika Yulia Darma, Warsiti, Heni Hendriyani, Nissa Prima Sari

**Affiliations:** a Department of Public Health and Community Medicine, Faculty of Medicine, Andalas University, Padang, Indonesia; b Department of Pharmacology, Faculty of Medicine, Andalas University, Padang, Indonesia; c Department of Midwifery, Syedza Saintika Institute of Health Science, Padang, Indonesia; d Department of Nursing, Faculty of Health Sciences, Aisyiyah University, Yogyakarta, Indonesia; e Department of Nutrition, Health Polytechnic, Semarang, Indonesia; f Department of Midwifery, Faculty of Medicine, Andalas University, Padang, Indonesia

**Keywords:** Breast cancer, Body mass index, High-fat diet, Indonesia, Reproductive, Risk Factors

## Abstract

This dataset describes a survey presenting reproductive, high-fat diet and body mass index (BMI) determinant factors for breast cancer among Indonesian women. The information was gathered from breast cancer and non-breast cancer patients via an online questionnaire, determining reproductive factors (menarche age, menopause age, first pregnancy age, parity, and breastfeeding), high-fat diet and BMI, from 1st June until 31th September 2020. Two hundred breast cancer patients and 200 non-breast cancer patients in Indonesia willing to fill out an online survey provided the samples. The data was analyzed using IBM version 25.0, which included univariate, bivariate, and multivariate analysis. The information would help Indonesian women in identifying the potential of breast cancer.

## Specifications Table

**Table:** 

Subject	Cancer epidemiology
Specific subject area	Cancer prevention, cancer screening
Type of data	Text, tables
How data were acquired	An online survey method was used to collect data (google forms). A supplementary file with the questionnaire is available.
Data format	Raw Analyzed
Parameters for data collection	The medical records of breast cancer and non-breast cancer patients at Sardjito General Hospital Yogyakarta and Dr. M. Djamil General Hospital Padang is reviewed. The data was collected from 200 women in Indonesia who had breast cancer and 200 women who did not have breast cancer to determining reproductive factors (menarche age, menopause age, first pregnancy age, parity, and breastfeeding), high-fat diet and BMI.
Description of data collection	An online survey was sent to breast cancer and non-breast cancer patients in Indonesia using a snowball method.
Data source location	Padang and Yogyakarta - Indonesia
Data accessibility	The data can be found on Mendeley Data. https://data.mendeley.com/datasets/xfcyrffhy7/2

## Value of the Data

• These findings are valuable since this is the first study of 400 participants, 200 of whom were breast cancer patients and 200 of whom were not that determine reproductive factors (menarche age, menopause age, first pregnancy age, parity, and breastfeeding), high-fat diet and BMI among Indonesian womens.
• These data can help the cancer registry, cancer epidemiology, and health promotion researchers because they can be used to improve community knowledge and understanding of breast cancer risk through early detection or screening. This information will help with health awareness, promotion, and education in order to reduce the high number of advanced-stage breast cancer populations.
• The set of data would be useful to investigators who want to equate their findings with related research from other countries on reproductive, high-fat diet, and BMI determinant factors for breast cancer, or who want to conduct a review study in the coming years.
• This information can have a community impact, measuring the risk will assist the public in providing routine breast cancer screenings and services for health in identifying patients at chance of developing breast cancer.

## 1 Data Description

According to the survey of Indonesian women's reproductive, high-fat diet, and BMI determinant factors for breast cancer, the dataset provides informative data. The medical records of breast cancer and non-breast cancer patients at Sardjito General Hospital Yogyakarta and Dr. M. Djamil General Hospital Padang is reviewed. The data was collected from 200 women in Indonesia who had breast cancer and 200 women who did not have breast cancer to determining reproductive factors: age of menarche (7–11 years old; 12–13 years old; > 13 years old) [1], Menopause age (≥ 50 years old; < 50 years old) [2], first pregnancy age (under 20 years old; between 20 and 29 years old; over 30 years old) [2], parity (nulliparous; primiparous; ≥ multiparous) [2], and breastfeeding (≥ 12 months; < 12 months) [3], [4]. The SQ-FFQ (Semi Quantitative Food Frequency Questionnaire) was utilised to assess diets high in fat (high, over 100 percent of RDA (Recommended Dietary Allowance); normal, 100 percent of RDA) [5] and BMI (normal, 18.5–23.49 kg/m^2^; overweight, 23.5–24.99 kg/m^2^; obesity, ≥ 25 kg/m^2^) [6]. The survey questions is available as a supplementary file. The patient demographics are presented in Table 1.

**Table 1:** Patient demographics (*n* = 400).

Demographics	Category	BC (*n* = 200) f (%)	Non-BC (*n* = 200) f (%)
**Age (years)**	< 50	95 (47.5)	84 (42.0)
	≥ 50	105 (52.5)	116 (58.0)
**Background in education**	No degree	3 (1.5)	1 (0.5)
	Primary school	26 (13.0)	19 (9.5)
	Middle school	16 (8.0)	19 (9.5)
	High school	81 (40.5)	78 (39.0)
	Bachelor’s diploma	68 (34.0)	79 (39.5)
	Graduate degree	6 (3.0)	4 (2.0)
**Working status**	Housewife	118 (59.0)	102 (51.0)
	Civil servant	60 (30.0)	16 (8.0)
	Private servant	15 (7.5)	66 (33.0)
	Enterpreneur	0	6 (3.0)
	Farmer	1 (0.5)	4 (2.0)
	Master’s student	0	2 (1.0)
	Retired	6 (3.0)	4 (2.0)
**Marital status**	Single/ widow	15 (7.5)	23 (11.5)
	Marriage	185 (92.5)	177 (88.5)

BC, breast cancer; non-BC, non-breast cancer.

Indonesian women’s reproductive, high-fat diet and BMI determinant factors for breast cancer is presented in Table 2. Table 3 described the determinant factors of breast cancer based on ethnicity stratification for Minangnese and Javanese people. Table 4 shows the unadjusted and adjusted OR as well as the 95% confidence intervals for breast cancer.

**Table 2:** Indonesian women’s reproductive, high-fat diet and BMI risk factors for breast cancer.

	Group		
Variables	BC (*n* = 200) f (%)	Non-BC (*n* = 200) f (%)	*p*-value
**Reproductive factors**			
Age of menarche (years)			0.600
7–11	18 (9.0)	16 (8.0)	
12–13	99 (49.5)	91 (45.5)	
> 13	83 (41.5)	93 (46.5)	
Age of menopause (years)			<0.001*^,^^a^
≥ 50	121 (60.5)	79 (39.5)	
< 50	79 (39.5)	121 (60.5)	
Age of the first pregnancy (years)			0.614
< 20	35 (17.5)	33 (16.5)	
20–29	137 (68.5)	147 (73.5)	
> 30	27 (13.5)	19 (9.5)	
Never been pregnant	1 (0.5)	1 (0.5	
Parity			0.097^a^
Nulliparous	1 (0.5)	2 (1.0)	
Primiparous	24 (12.0)	39 (19.5)	
≥ Multiparous	175 (87.5)	159 (79.5)	
Breastfeeding			<0.001*^,^^a^
≥ 12 months	198 (99.0)	157 (78.5)	
< 12 months	2 (1.0)	43 (21.5)	
**High-fat diet**			<0.001*^,^^a^
High	192 (96.0)	88 (44.0)	
Normal	8 (4.0)	112 (56.0)	
**BMI**			0.001*^,^^a^
Normal	87 (43.5)	125 (62.5)	
Overweight	38 (19.0)	21 (10.5)	
Obesity	75 (37.5)	54 (27.0)	

⁎ , significant at *p* < 0.05.

a , *p* < 0.25 entered the logistic regression; BMI, body mass index.

**Table 3:** The determinant factors of breast cancer based on ethnicity stratification for minangnese and javanese people.

		Minangnese (n-200)		Javanese (*n* = 200)		
Variables	Category	BC (f/%)	Non-BC (f/%)	BC (f/%)	Non-BC (f/%)	p-value
Age of menarche	7–11 years	10 (10.0)	8 (8.0)	8 (8.0)	8 (8.0)	<0.001*
	12–13 years	52 (52.0)	49 (49.0)	47 (47.0)	42 (42.0)	ref
	> 13 years	38 (38.0)	43 (43.0)	45 (45.0)	50 (50.0)	<0.001*
Age of menopause	<50 (years)	37 (37.0)	63 (63.0)	42 (42.0)	58 (58.0)	ref
	≥ 50 (years)	63 (63.0)	37 (37.0)	58 (58.0)	42 (42.0)	<0.001*
Age of the first pregnancy	20–29 (years)	65 (65.0)	65 (65.0)	72 (72.0)	82 (82.0)	ref
	<20 (years)	19 (19.0)	22 (22.0)	16 (16.0)	11 (11.0)	<0.001*
	≥ 30 (years)	15 (15.0)	13 (13.0)	12 (12.0)	6 (6.0)	<0.001*
	Never	1 (1.0)	0	0	1 (1.0)	0.002*
Parity	Nulliparous	1 (1.0)	1 (1.0)	0	1 (1.0)	ref
	Primiparous	13 (13.0)	20 (20.0)	11 (11.0)	19 (19.0)	<0.001*
	≥ Multiparous	86 (86.0)	79 (79.0)	89 (89.0)	80 (80.0)	ref
Breastfeeding	≥ 12 months	99 (99.0)	75 (75.0)	99 (99.0)	82 (82.0)	<0.001*
	< 12 month	1 (1.0)	25 (25.0)	1 (1.0)	18 (18.0)	ref
High-fat diet	Normal	6 (6.0)	51 (51.0)	2 (2.0)	61 (61.0)	ref
	High	94 (94.0)	49 (49.0)	98 (98.0)	39 (39.0)	<0.001*
BMI	Normal	44 (44.0)	66 (66.0)	43 (43.0)	59 (59.0)	ref
	Overweight	20 (20.0)	9 (9.0)	18 (18.0)	12 (12.0)	<0.001*
	Obesity	36 (36.0)	25 (25.0)	39 (39.0)	29 (29.0)	<0.001*

⁎ , ref, reference; In the Mantel-Haenszel test, the homogeneity was *p* < 0.05.

**Table 4:** The unadjusted and adjusted OR for breast cancer.

		Unadjusted OR		Adjusted OR	
Variables	Category	OR (95%CI)	p-value	OR (95%CI)	*p*-value
Age of menopause	<50 (years)	ref	ref	ref	ref
	≥ 50 (years)	2.35 (1.57–3.50)	<0.001*	1.98 (1.18–3.35)	0.010*
Parity	Nulliparous	0.81 (0.07–9.45)	0.868	0.31 (0.01–6.63)	0.307
	Primiparous	ref	ref	ref	ref
	≥ Multiparous	1.78 (1.03–3.11)	0.039*	1.04 (0.47–2.31)	0.918
Breastfeeding	< 12 months	ref	ref	ref	ref
	≥ 12 months	27.12 (6.47–113.66)	<0.001*	19.94 (4.26–93.27)	<0.001*
High-fat diet	Normal	ref	ref	ref	ref
	High	30.55 (14.28–65.34)	<0.001	33.08 (14.85–73.71)	<0.001*
BMI	Normal	ref	ref	ref	ref
	Overweight	2.60 (1.43–4.73)	0.002*	3.33 (1.46–7.61)	0.004*
	Obesity	1.99 (1.28–3.11)	0.002*	2.53 (1.39–4.60)	<0.001*

OR, odd ratio; ref, reference.

⁎ , significant at *p* < 0.05.

## 2 Experimental Design, Materials and Methods

This dataset used a case-control method to assess the reproductive, high-fat diet, and BMI determinant factors for breast cancer in Indonesian females. The dataset consisted of 200 breast cancer patients and 200 non-breast cancer patients gathered via medical documents check at Sardjito General Hospital Yogyakarta and Dr. M. Djamil General Hospital Padang, as well as written informed consent via the internet. At the start of the questionnaire, all participants were told that their responses would only be used for survey purposes. The participation of eligible subjects in this survey was entirely voluntary. On the first page of the survey, electronic informed consent was shown. The data responses were obtained between June 1st and September 30th, 2020. For recruiting potential participants, the principal investigators chose WhatsApp Messenger. Following permission from physicians or members of a team who cared for patients at Sardjito Hospital Yogyakarta and Dr. M Djamil Hospital Padang, the principal researchers received the phone numbers of participants from medical documents analysis, a questionnaire was developed, implemented, and distributed on Whatsapp messenger. In this dataset, convenience sampling was used as the sampling process [7]. Female patients with pathology exams confirming positive breast cancer based on medical documents analysis, as well as non-breast cancer and non-ovarian cancer patients, were included [8]. Male breast cancer survivors are the exclusion criterion for all breast cancer and non-breast cancer patients categories. Based on hospital control, the non-breast cancer group was matched for sex and age by 5 years.

The bivariate analysis was performed using test of chi-square with a *p* < 0.05 was declared significant. The variable that was declared statistically significant as a potential variable with a *p* < 0.25 was continued with logistic regression. The Mantel-Haenszel test for breast cancer risk is focused on grouping by ethnicity (Minangnese and Javanese). In the Mantel-Haenszel test, P value 0.05 homogeneous. The relationship between women’s reproductive, high-fat diets, and BMI for breast cancer risk was analyzed using logistic regression analysis, and the analysis were shown as OR with 95% CI. Data analysis used the IBM version 25.0.

## Ethics Statement

The committee on ethics of the Faculty of Medicine, Andalas University, Padang, Indonesia (No. 342/ KEP/ FK/ 2020) approved this report. The survey followed the Helsinki Declaration's standards.

## CRediT Author Statement

**Ricvan Dana Nindrea:** conceptualization, methodology, formal analysis, visualisation, writing original draft; **Elly Usman:** investigation, methodology, writing original draft; **Yusticia Katar:** investigation, and methodology; **Ika Yulia Darma:** formal analysis, and methodology; **Warsiti:** data curation, and investigation; **Heni Hendriyani:** data curation, investigation, methodolology; **Nissa Prima Sari:** investigation, and writing-review.

## Declaration of Competing Interest

The authors claim that they have no known financial or individual interests that could have influenced the survey presented in this dataset.

## Appendix Supplementary materials

Image, application 1

## References

[1] (2012). Collaborative group on hormonal factors in breast cancer, menarche, menopause, and breast cancer risk: individual participant meta-analysis, including 118 964 women with breast cancer from 117 epidemiological studies. Lancet. Oncol..

[2] Chen H.L., Zhou M.Q., Tian W., Meng K.X., He H.F. (2016). Effect of age on breast cancer patient prognoses: a population-based study using the SEER 18 database. PloS One.

[3] (2002). Collaborative group on hormonal factors in breast cancer, breast cancer and breastfeeding: collaborative reanalysis of individual data from 47 epidemiological studies in 30 countries, including 50302 women with breast cancer and 96973 women without the disease. Lancet.

[4] Nindrea R.D., Aryandono T., Lazuardi L. (2017). Breast cancer risk from modifiable and non-modifiable risk factors among women in Southeast Asia: a meta-analysis. Asian. Pac. J. Cancer. Prev.

[5] Ministry of Health Republic of Indonesia (2013). Basic Health Research in 2013.

[6] Expert Consultation WHO (2004). Appropriate body-mass index for Asian populations and its implications for policy and intervention strategies. Lancet.

[7] Nindrea R.D., Sari N.P., Harahap W.A., Haryono S.J., Kusnanto H., Dwiprahasto I. (2020). Survey data of COVID-19 awareness, knowledge, preparedness and related behaviors among breast cancer patients in Indonesia. Data Brief.

[8] Nindrea R.D., Sari N.P., Harahap W.A., Haryono S.J., Kusnanto H., Dwiprahasto I. (2020). Survey data of multidrug-resistant tuberculosis,Tuberculosis patients characteristics and stress resilience during COVID-19 pandemic in West Sumatera province, Indonesia.

