# Supplementary material for: Dataset of Indonesian women’s reproductive, high-fat diet and body mass index risk factors for breast cancer
Source: Data Brief. 2021 Apr 29;36:107107. doi: 10.1016/j.dib.2021.107107 (PMC8134712; doi:10.1016/j.dib.2021.107107)
Supplement: Supplementary file 1 [file mmc1.docx]

**Questionnaire**

**Research on Indonesian women’s reproductive, high-fat diet and body mass index risk factors for breast cancer**

Assalamu Alaikum

I would like to thank you for taking your valuable time to complete our survey on **Indonesian women’s reproductive, high-fat diet and body mass index risk factors for breast cancer**. The information you are about to provide is for research purposes only. The survey will take approximately 5 -10 minutes to finish. All of your responses will be kept strictly confidential and never disclosed your name. Your help is greatly appreciated.

If you have any questions about how to complete this questionnaire or if you have any additional comments or concerns you: would like to share regarding this survey, please feel free contact us.

Dr. Ricvan Dana Nindrea

Nissa Prima Sari, M.Sc

*Required

**Section A: Patient characteristics**

1. Age*

Mark only one square

|  | <50 years |
| --- | --- |
|  | ≥ 50 years |

1. Educational background*

Mark only one square

|  | No school |
| --- | --- |
|  | Elementary school |
|  | Junior high school |
|  | Senior high school |
|  | Bachelor degree |
|  | Master degree |

1. Working status*

Mark only one square

|  | Housewife |
| --- | --- |
|  | Civil servant |
|  | Private servant |
|  | Enterpreneur |
|  | Farmer |
|  | Master’s student |
|  | Retired |

1. Marital status*

Mark only one square

|  | Single/ widow |
| --- | --- |
|  | Marriage |

**Section B: Reproductive factors**

1. Age of menarche*

Mark only one square

|  | 7-11 years |
| --- | --- |
|  | 12-13 years |
|  | > 13 years |

1. Age of menopause*

Mark only one square

|  | ≥ 50 years |
| --- | --- |
|  | < 50 years |

1. Age of the first pregnancy*

Mark only one square

|  | < 20 years |
| --- | --- |
|  | 20-29 years |
|  | > 30 years |
|  | Never been pregnant |

1. Parity*

Mark only one square

|  | Nulliparous |
| --- | --- |
|  | Primiparous |
|  | ≥ Multiparous |

1. Breastfeeding*

Mark only one square

|  | ≥ 12 months |
| --- | --- |
|  | < 12 months |

**Section C: High-fat diet**

1. High-fat diet*

Mark only one square

|  | High (excess, >100% Recommended Dietary |
| --- | --- |
|  | Normal (sufficient, 100% RDA) |

**Section D: Body mass index**

1. Body mass index*

Mark only one square

|  | Normal, 18.5-23.49 kg/m2 |
| --- | --- |
|  | Overweight, 23.5-24.99 kg/m2 |
|  | Obesity, ≥25 kg/m2 |
